# Resistance to topoisomerase cleavage complex induced lethality in *Escherichia coli *via titration of transcription regulators PurR and FNR

**DOI:** 10.1186/1471-2180-11-261

**Published:** 2011-12-12

**Authors:** I-Fen Liu, Sandra Aedo, Yuk-Ching Tse-Dinh

**Affiliations:** 1 Department of Biochemistry and Molecular Biology, New York Medical College, Valhalla, NY 10595, USA

## Abstract

**Background:**

Accumulation of gyrase cleavage complex in *Escherichia coli *from the action of quinolone antibiotics induces an oxidative damage cell death pathway. The oxidative cell death pathway has also been shown to be involved in the lethality following accumulation of cleavage complex formed by bacterial topoisomerase I with mutations that result in defective DNA religation.

**Methods:**

A high copy number plasmid clone spanning the *upp-purMN *region was isolated from screening of an *E. coli *genomic library and analyzed for conferring increased survival rates following accumulation of mutant topoisomerase I proteins as well as treatment with the gyrase inhibitor norfloxacin.

**Results:**

Analysis of the intergenic region upstream of *purM *demonstrated a novel mechanism of resistance to the covalent protein-DNA cleavage complex through titration of the cellular transcription regulators FNR and PurR responsible for oxygen sensing and repression of purine nucleotide synthesis respectively. Addition of adenine to defined growth medium had similar protective effect for survival following accumulation of topoisomerase cleavage complex, suggesting that increase in purine level can protect against cell death.

**Conclusions:**

Perturbation of the global regulator FNR and PurR functions as well as increase in purine nucleotide availability could affect the oxidative damage cell death pathway initiated by topoisomerase cleavage complex.

## Background

DNA topoisomerases catalyze topological transformations of DNA by concerted breaking and rejoining of DNA strands via the formation of a covalent complex between the enzyme and cleaved DNA [1]. While the activities of topoisomerases are critical for vital cellular functions, topoisomerase enzymes are also vulnerable targets for cell killing because DNA rejoining by topoisomerases can often be inhibited by antibacterial or anticancer agents that are referred to as topoisomerase poisons [2,3]. Quinolones are widely used antibacterial drugs that lead to the accumulation of covalent cleavage complex formed by the bacterial type IIA topoisomerases, DNA gyrase and topoisomerase IV [4,5]. The accumulation of DNA gyrase covalent complex from the action of quinolones has been shown to induce an oxidative damage cell death pathway in *E. coli *as at least one of the potential mechanisms of cell killing [6-9]. The sequence of events following topoisomerase cleavage complex accumulation that leads to generation of reactive oxygen species remains unclear.

Although a specific poison for bacterial topoisomerase I remains to be identified, accumulation of topoisomerase I cleavage complex in *E. coli *has also been shown to lead to rapid cell death from the study of topoisomerase I mutants defective in DNA rejoining [10,11]. Similar to gyrase cleavage complex, topoisomerase I cleavage complex accumulation in *E. coli *induces the SOS response via the RecBCD pathway [12]. Increase in reactive oxygen species has been shown to also contribute to the cell death pathway initiated by accumulation of topoisomerase I cleavage complex [13]. Recombinant *E. coli *and *Yersinia pestis *topoisomerase I mutants that accumulate the covalent cleavage complex due to deficiency in DNA rejoining provide useful model systems for studying the physiological effect of topoisomerase-DNA cleavage complex accumulation. *Y. pestis *topoisomerase I (YpTOP1) is highly homologous to *E. coli *topoisomerase I, with the advantage of its dominant lethal recombinant clones being more stable in *E. coli *than comparable *E. coli *topoisomerase I mutant clones. The *Y. pestis *mutant topoisomerase I model system has been utilized to screen for *E. coli *genomic clones, that when present in high copy number on a plasmid, can confer resistance to topoisomerase cleavage complex induced cell killing. Additional experiments on an isolated clone demonstrated a novel mechanism of increased resistance to topoisomerase cleavage complex via titration of the transcription factors FNR and PurR by a high copy number plasmid clone of the intergenic region between *upp *and *purM*. This plasmid clone also increased bacterial resistance to norfloxacin that induces the accumulation of the type IIA topoisomerase covalent cleavage complex. FNR regulates transition between anaerobic and aerobic conditions [14,15]. Genome-wide expression analysis has previously shown that FNR contributes to the repression of a number of genes induced by oxidative stress conditions [16,17]. PurR is a suppressor of purine biosynthesis. Titration of the FNR and PurR transcription factors by the high copy number clone is expected to increase the expression level of genes normally suppressed by these two regulators. These results provide further insights into the oxidative cell death pathways initiated by topoisomerase cleavage complex accumulation.

## Results

### Isolation of clone pAQ5 containing the *upp-purMN *region in selection for resistance to topoisomerase I cleavage complex mediated cell death

**Figure 1:**
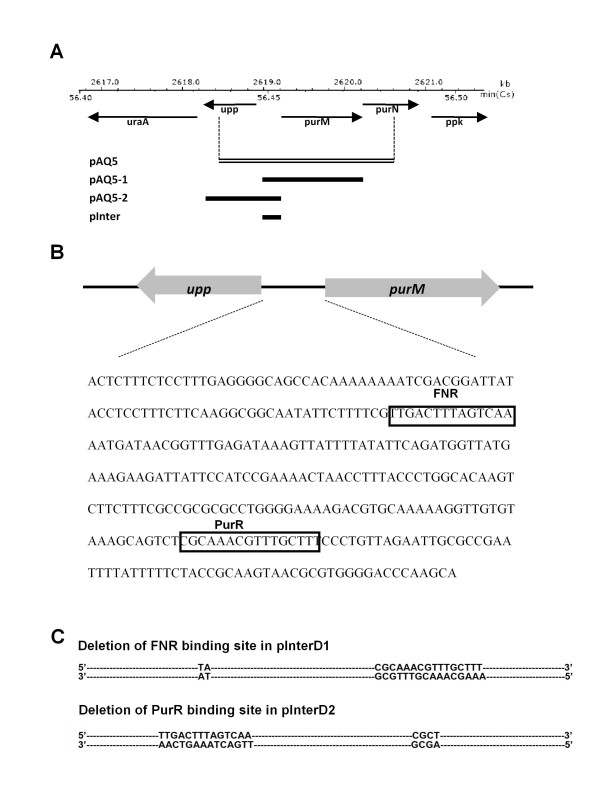
***E. coli *chromosomal DNA insert in high copy plasmid clone pAQ5 and its derivatives (**a**) Clone pAQ5 containing sequence in the *upp-purM-purN *region was selected from an *E. coli *genomic DNA plasmid library for resistance to cell killing mediated by mutant topoisomerase I YpTOP1-D117N expressed in BW117N**. PCR was used to amplify the intergenic sequence shown in (**b**) for cloning into pCR-TOPO-XL cloning vector in the construction of pInter. The sequence of the FNR and PurR binding site deleted in pInterD1 and pInterD2 is shown in (**c**).

**Figure 2:**
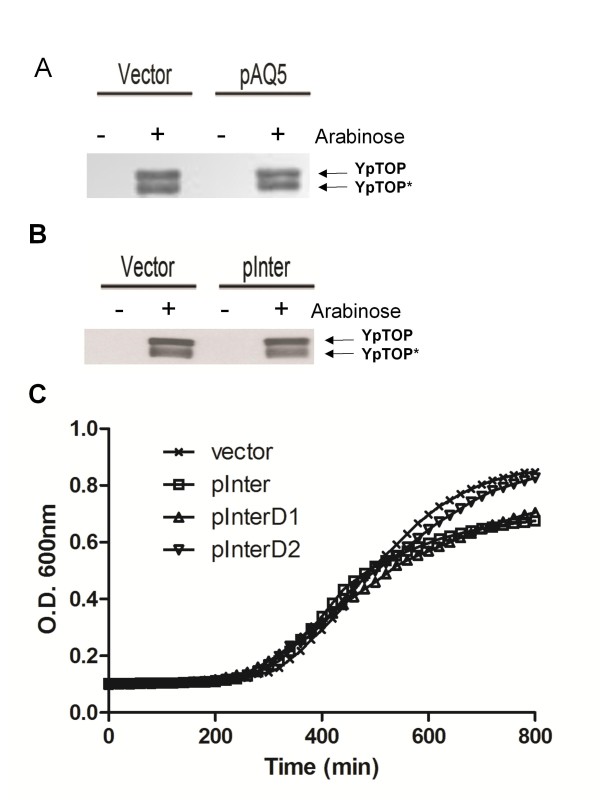
**Effect of plasmid clones on recombinant mutant *Y. pestis *topoisomerase I expression and growth rates Western blot analysis of expression of mutant *Y. pestis *topoisomerase I in the presence of control vector and clone pAQ5 (a) or pInter (b)**. Exponential phase cultures were treated with 0.002% arabinose for 2.5 h. Total cellular protein was analyzed by SDS PAGE and Western blot with mouse monoclonal antibodies against *E. coli *topoisomerase I (EcTOP). This antibody recognizes the highly homologous *Y. pestis *topoisomerase I (YpTOP) and its partially degraded product (YpTOP*).(**C**) Growth of BW27784 transformed with vector, pInter, pInterD1 and pInterD2 in LB. Absorbance was measured in a 96 well microplate at 37°C every 20 min using the Perkin Elmer 7000 Plus BioAssay Reader with the filter set at 590 nm and shaking for 10 min before each measurement.

After transformation of *E. coli *strain BW117N with the *E. coli *genomic DNA library generated with the pCR-XL-TOPO cloning system, four different plasmid clones isolated from colonies obtained on LB plates with 0.002% arabinose were confirmed to increase resistance to the dominant lethal effect of the mutant *Y. pestis *topoisomerase I, YpTOP1-D117N [10]. Detailed analysis of the clone pAQ5 containing the *upp-purMN *region of *E. coli *chromosome (corresponding to nucleotides 2618398-2620765 of *E. coli *MG1655 sequence, Figure 1a) is described here. Strain BW117N is under strong selective pressure to eliminate expression of the dominant lethal mutant YpTOP1-D117N. Subsequent analysis of the effect of clone pAQ5 or its derivatives was therefore carried out with strain BW27784 carrying plasmid pAYTOP128 expressing YpTOP1 with the less lethal G122S mutation that also leads to accumulation of the topoisomerase I cleavage complex [11]. Clone pAQ5 was found to increase survival following arabinose induction of this mutant YpTOP1 by 63-fold compared to the control empty vector (Table 1). This clone (Figure 1a) contains the entire *purM *(5'-phosphoribosyl-5-aminoimidazole synthetase) coding sequence (2619219-2620256), part of the *purN *(phosphoribosylglycinamide formyltransferase) coding sequence (2620256-2620894), and part of the *upp *(uracil phosphoribosyltransferase) coding sequence (2618268-2618894), plus the intergenic regulatory region between the *upp *and *purMN *genes (2618946-2619178). Analysis of total cellular protein by western blot showed that clone pAQ5 did not confer resistance by decreasing the expression level of mutant topoisomerase I after arabinose induction (Figure 2a).

**Table 1:** Effect of high copy plasmid clones on survival following accumulation of mutant topoisomerase I cleavage complex

Plasmid	Survival Ratio
pCRII vector	7.85 × 10^-5 ^± 1.19 × 10^-5^

pAQ5	4.95 × 10^-3 ^± 1.55 × 10^-3^

pAQ5-1	4.92 × 10^-3 ^± 1.20 × 10^-3^

pAQ5-2	1.25 × 10^-2 ^± 2.48 × 10^-3^

pInter	1.90 × 10^-2 ^± 4.12 × 10^-3^

pInterD1	4.22 × 10^-3 ^± 1.02 × 10^-3^

pInterD2	5.19 × 10^-4 ^± 1.73 × 10^-4^

*E. coli *BW27784 carrying pAYTOP128 was transformed with high copy number plasmid shown in the table. Cultures were grown to exponential phase with shaking, then treated with 0.002% arabinose for 2.5 h before serial dilution and plating on LB plates with antibiotics and 2% glucose

Survival ratio was determined by calculating the ratio of the viable colony counts obtained from the induced cultures versus the viable counts from non-induced culture. The results represent the average and standard errors from at least three experiments

### Analysis of resistance to topoisomerase I cleavage complex conferred by *upp-purMN *intergentic region

To determine the basis of resistance from clone pAQ5, derivatives of pAQ5 were constructed by cloning of specific PCR amplified DNA into pCR-XL-TOPO vector. These include clones pAQ5-1with *purM *and the intergenic region, pAQ5-2 with *uppA *and the intergenic region, and pInter, with the intergenic region alone (Figure 1a). These clones were transformed into strain BW27784 containing pAYTOP128 expressing mutant *Y. pestis *topoisomerase I deficient in DNA religation due to the TOPRIM G122S mutation to investigate the effect of the clones on viability following induction of mutant topoisomerase I. The results (Table 1) showed that the intergenic region alone in clone pInter was sufficient to confer resistance to the mutant topoisomerase I. Western blot analysis confirmed that the protective effect of pInter was also not due to reduction in expression level of mutant topoisomerase I (Figure 2b).

Examination of this intergenic sequence showed that it includes the binding site sequences of two transcription factors, FNR and PurR (Figure 1b). The FNR binding sequence, TTGACTTTAGTCAA versus the TTGATN_4_ATCAA consensus sequence [18-20], is located 61.5 nucleotides upstream of the *upp *transcription start site. The PurR binding sequence, CGCAAACGTTTGCTT, versus the consensus PurR operator sequence of CGCAAACGTTTNCNT [21], is located 28 nucleotides upstream of the *purM *gene. FNR acts as a dual transcription regulator that activates certain genes required for anaerobic growth and represses many genes required for aerobic growth [22]. Its interaction with the *upp-purMN *region has been reported previously [19]. PurR negatively regulates the transcription of genes involved in purine and pyrimidine nucleotide synthesis including *purMN *[21,23,24]. We therefore hypothesize that the high copy number pInter could titrate these transcription factors to relieve the repression of other *E. coli *genes encoded on the chromosome. To test this hypothesis, these binding sites were eliminated individually by site-directed mutagenesis (Figure 1c). Nucleotides TGACTTTAGTCA were deleted from the FNR binding site to result in plasmid pInterD1. Nucleotides AAACGTTTGCTT were deleted from the PurR binding site to result in plasmid pInterD2. Measurement of cell viability following induction of mutant topoisomerase from pAYTOP128 showed that elimination of either of these two binding sites reduced the protective effect of pInter, (Table 1). Comparison of the growth curves of these strains (Figure 2c) showed that while cells transformed with pInter and pInterD1 grew to a lower density at saturation, the initial growth rates of these strains are similar. The slightly slower growth rate of cells transformed with pInterD1 was not statistically significant and since pInterD1 conferred a lesser degree of resistance than pInter, the difference in viability following accumulation of topoisomerase I cleavage complex cannot be accounted for simply as due to growth inhibition.

Effect of high copy number plasmid clone pInter on sensitivity to norfloxacin BW27784 transformed with the high copy number plasmid clones pAQ5 or pInter were treated with the gyrase inhibitor norfloxacin to determine if the plasmids could confer resistance also to cell death mediated by type II topoisomerase cleavage complex. The results (Table 2) showed that these plasmids could confer ~30-fold higher survival rates than the control vector. Deletion of either the FNR or PurR binding site from pInter decreased the protective effect, demonstrating that titration of these transcription factors could result in increased resistance to norfloxacin. It should be noted that pInterD1 conferred more protection than pInterD2 to mutant topoisomerase I killing (Table 1) and the opposite was true for norfloxacin killing (Table 2).

**Table 2:** Effect of high copy plasmid clones on survival following treatment with norfloxacin

Plasmid	Survival Ratio
pCRII vector	2.14 × 10^-5 ^± 4.1 × 10^-6^

pAQ5	7.57 × 10^-4 ^± 2.14 × 10^-4^

pInter	6.12 × 10^-4 ^± 1.28 × 10^-4^

pInterD1	8.41 × 10^-5 ^± 3.55 × 10^-5^

pInterD2	1.11 × 10^-4 ^± 2.01 × 10^-5^

*E. coli *BW27784 transformed with high copy number plasmid was grown to exponential phase with shaking. Cultures were treated with 250 ng/ml norfloxacin for 2 h before serial dilution and plating on LB plates with kanamycin

Survival ratio was determined by calculating the ratio of the viable colony counts obtained from the treated cultures versus the viable counts from untreated culture. The results represent the average and standard errors from at least three experiments

### Protective effect from adenine addition

**Figure 3:**
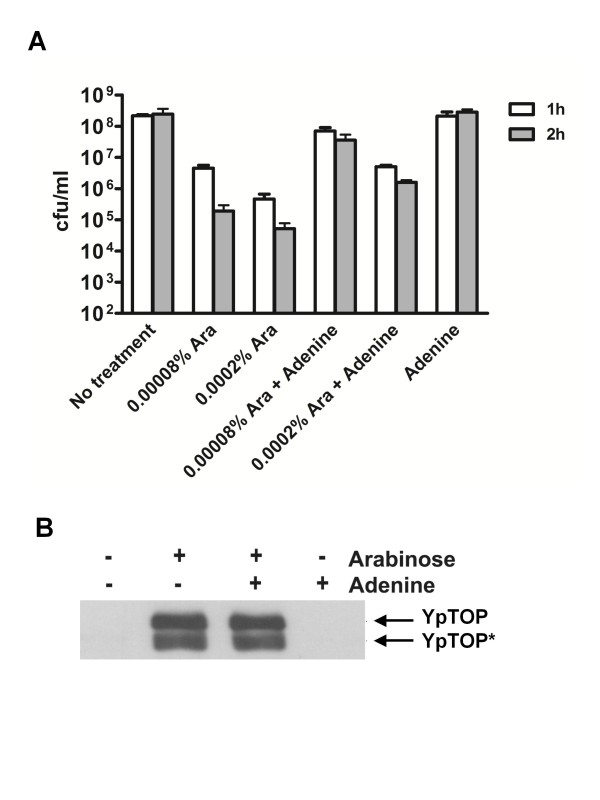
**Addition of adenine to minimal medium increases survival following induction of mutant topoisomerase I cleavage complex BW27784 transformed with pAYTOP128 was grown overnight in RM minimal medium with 2% glucose to suppress mutant topoisomerase I expression, then diluted 1:100 into RM medium with 0.2% glycerol**. When OD_600 _reached 0.4, 0.00008% or 0.0002% arabinose was added with or without 100 μg/ml adenine included. Viable colony counts were determined at 1 h and 2 h after arabinose addition (**a**). The presence of adenine did not affect expression of mutant YpTOP after induction of 0.0002% arabinose for 2 h as analyzed by Western blot (**b**).

The protective effect from titration of PurR could be due to increased availability of purine nucleotides. This was tested by growth of BW27784 transformed with pAYTOP128 in minimal media. Greater than 3 logs of loss of viability could be measured at 2 h after induction of mutant topoisomerase I expression by 0.0002% arabinose (Figure 3a). The presence of 100 μg/ml adenine in the growth medium increased the number of viable colonies by 30-fold at 2 h after arabinose addition. The presence of adenine did not affect expression level of mutant topoisomerase I as determined by western blot (Figure 3B).

**Figure 4:**
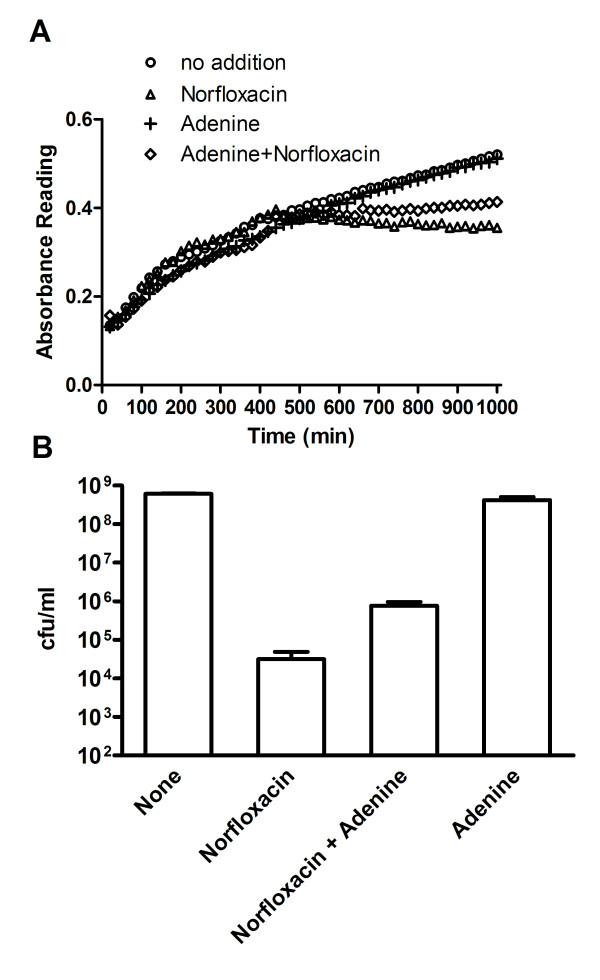
**Decreased sensitivity to norfloxacin from addition of adenine BW27784 was grown overnight in RM minimal medium with 0.2% glycerol and then diluted 1:100 and grown to exponential phase**. (**a**) The exponential phase culture was diluted twofold with RM medium with (o) no addition, (**+**) 250 μg/ml adenine, (Δ)120 ng/ml norfloxacin, or (◊)120 ng/ml norfloxacin and 250 μg/ml adenine. Absorbance was measured at 37°C every 20 min using the Perkin Elmer 7000 Plus BioAssay Reader with the filter set at 590 nm and shaking for 10 min before each measurement. (**b**) Exponential phase culture was treated with 200 ng/ml norfloxacin with or without 250 μg/ml adenine along with controls with no treatment or adenine alone. After 3 h at 37°C, viable colony counts were determined by dilution and plating on LB plates.

To determine if addition of adenine affects sensitivity to norfloxacin, BW27784 cells grown in minimal medium with different adenine concentrations were first evaluated by examining growth inhibition by norfloxacin. Increased resistance to growth inhibition by norfloxacin was observed in the presence of 250 μg/ml adenine (Figure 4a). Growth of BW27784 in the absence of norfloxacin was not affected significantly by the presence of adenine. Viable colony counts at 3 h after norfloxacin treatment were then measured and found to be increased 24-fold by the presence of adenine (Figure 4b).

### The high copy number intergenic region clone decreases the level of hydroxyl radicals following norfloxacin treatment

**Figure 5:**
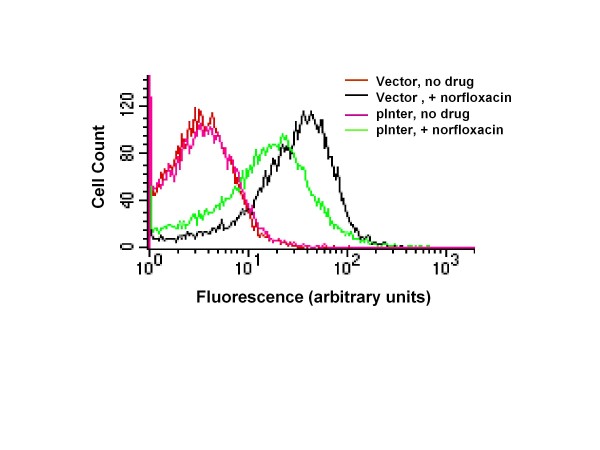
**The presence of pInter decreased the level of hydroxyl radicals present in norfloxacin-treated cells *E. coli *BW27784 with control vector or pInter were grown to exponential phase before treatment with 250 ng/ml norfloxacin**. HPF was added 2 h later for fluorescence detection of hydroxyl radicals by flow cytometry. The results represent a single experiment out of four independent experiments (p < 0.05 for decrease in fluorescence after norfloxacin treatment due to the presence of pInter).

The high copy number pInter resulted in ~30-fold higher ratio of viability after treatment with norfloxacin when compared to control plasmid with no insert (Table 2). Bactericidal antibiotics have been shown to initiate formation of reactive oxygen species in their cell killing mechanism [7,8,25], and hydroxyl radicals formation has been shown to be involved in bacterial cell death following topoisomerase I cleavage accumulation [13]. We hypothesize that the high copy number of the *upp-purMN *intergenic region modulates cellular metabolism to reduce the formation of reactive oxygen species upon accumulation of topoisomerase I cleavage complex. Formation of hydroxyl radicals was followed by increase in fluorescence intensity from reporter HPF [7]. The results (Figure 5) showed that at 2 h after addition of 250 ng/ml of norfloxacin, HPF fluorescence intensity from hydroxyl radicals in BW27784 cells transformed with pInter was reduced compared to HPF fluorescence from BW27784 transformed with vector after drug treatment.

### Effect of chromosomal *fnr *and *purR *mutations on sensitivity to topoisomerase I cleavage complex accumulation

**Figure 6:**
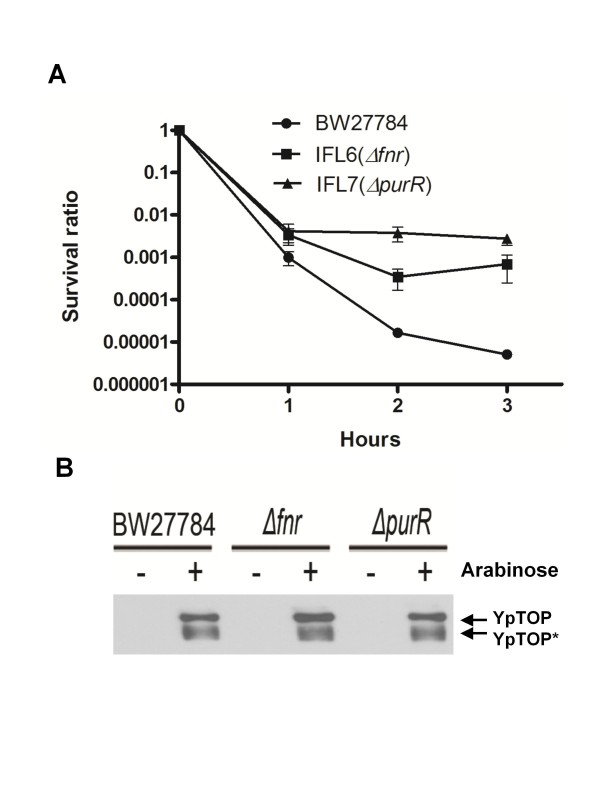
**Effect of Δ*purR *and Δ*fnr *mutations on sensitivity to topoisomerase I cleavage complex Strains BW27784 and its isogenic derivatives IFL6 (Δ*fnr*), IFL7 (*ΔpurR*) were transformed with pAYTOP128 and grown in LB with shaking to exponential phase before the addition of 0.002% arabinose**. (**a**) Viable colony counts of arabinose treated cultures were divided by the colony counts from the untreated culture to obtain the survival ratio. (**b**) Western blot analysis showed that the *ΔpurR *and *Δfnr *mutations did not affect expression levels of mutant YpTOP after induction with 0.002% arabinose for 2.5 h.

To support the hypothesis that the protective effect from pInter is due to the titration of the transcription factors FNR and PurR, chromosomal mutations eliminating the activity of the *fnr *and *purR *genes were introduced into BW27784 by P1 transduction resulting in strains IFL6 *(Δfnr*) and IFL7 *(ΔpurR*). Viable colony counts were measured following induction of mutant topoisomerase I expression from pAYTOP128. The results showed that in agreement with the hypothesis, higher rates of survivals were observed in the absence of FNR or PurR activity, with a greater effect from the *purR *mutation (Figure 6a). Western blot analysis was used to confirm that the mutations did not affect induction of mutant topoisomerase I expression (Figure 6b). It is consistent that PurR loss, either by *purR *mutation shown in Figure 6a or titration by high copy of its binding site in pInterD1 and other plasmids shown in Table 1, increases resistance.

### The protective effect of *Δfnr *mutation was greater under low oxygen conditions

The genes suppressed by FNR directly or indirectly via the sRNA FnrS [26,27] include many aerobic metabolic genes as well as genes involved in removal of reactive oxygen species such as *katE, sodA *and *sodB *[16]. Hydroxyl radicals generated from superoxide have been shown to be involved in the cell killing pathway initiated by topoisomerase I cleavage complex [13]. Cells in liquid cultures have been incubated with shaking at 215 rpm in our experiments carried out so far. Gene regulation by FNR is responsive to low level of oxygen [22,28]. We therefore modified our experimental conditions to decrease oxygen availability. BW27784 or IFL6 *(Δfnr*) cells were grown without shaking in a closed vessel until OD_600 _= 0.4. After addition of arabinose to induce mutant topoisomerase I expressed by pAYTOP128, the culture was divided into two portions and incubation was continued with and without shaking. Measurement of survival ratio (ratio of viable colonies compared to control culture with no arabinose added) shown in Table 3 indicated that for BW27784, the survival ratio after induction of mutant topoisomerase I is higher in culture without shaking (around tenfold), likely due to lower level of reactive oxygen species. The protective effect from the *Δfnr *mutation was more prominent when oxygen was limiting versus when oxygen was available. This is in agreement with the active role of FNR in gene regulation under anaerobic conditions.

**Table 3:** Protective effect of *Δfnr *mutation for cell killing initiated by mutant topoisomerase I cleavage complex accumulation under aerobic and low oxygen conditions

Survival Ratio		
	**Aerobic**	**Low Oxygen**

BW27784	1.18 × 10^-4 ^± 7.7 × 10^-5^	1.07 × 10^-3 ^± 4.7 × 10^-4^

IFL6	1.30 × 10^-3 ^± 3.1 × 10^-4^	8.15 × 10^-2 ^± 3.1 × 10^-3^

*Δfnr *effect	11-fold	76-fold

Cultures were grown to exponential phase without shaking, then treated with 0.002% arabinose for 2.5 h under either aerobic or low oxygen conditions before serial dilution and plating on LB plates with antibiotics and 2% glucose. Survival ratio was determined by calculating the ratio of the viable colony counts obtained from the induced cultures versus the viable counts from non-induced culture. The results represent the average and standard errors from at least three experiments

However, chromosomal *ΔpurR *and *Δfnr *mutations were found to have little effect on the viable colony counts at 1 and 2 h after treatment with up to 250 ng/ml norfloxacin (data not shown). Greater than 1000-fold lower bactericidal rates were observed for BW27784 with oxygen limitation when compared to incubation with oxygen after treatment with norfloxacin, in agreement with previous report of decreased norfloxacin sensitivity under anaerobic conditions [29]. It is therefore not feasible to investigate any potential protective effect from pInter or the *Δfnr *mutation under low oxygen conditions.

## Discussion

A segment of *E. coli *chromosomal DNA spanning the *upp-purMN *region was selected from a high copy number plasmid library of *E. coli *genomic DNA fragments based on its ability to confer resistance to cell killing mediated by accumulation of topoisomerase I cleavage complex. The intergenic region of *upp-purMN *was found to protect against bacterial cell death initiated by both type I and type II covalent topoisomerase-DNA cleavage complex. Deletion of the binding sites for FNR and PurR decreased the protective effect, suggesting that the protective effect we observed for pInter resulted from titration of the transcription regulators FNR and PurR.

PurR is a repressor of purine biosynthesis in *E. coli *[19]. The hypothesis that the protective effects observed from the high copy number plasmid pInter is related to purine nucleotide pool availability is supported by the increased viability when adenine was added to defined medium. The *ΔpurR *mutation resulted in up to 475-fold higher survival rate following topoisomerase I covalent cleavage complex accumulation. Although pInter could increase survival rate following norfloxacin treatment, the *ΔpurR *chromosomal mutation did not affect norfloxacin sensitivity. Deletion mutation of a global transcription regulator is likely to affect the many metabolic genes under its regulation differently than titration of the global transcription regulator by the presence of its binding site on a high copy number plasmid. Chromosomal PurR recognition sites with the strongest binding affinity for PurR might still be repressed by PurR even in the presence of pInter but they would be depressed in the *ΔpurR *background. The cell death pathways initiated by type IA and type IIA topoisomerases may be affected to different degrees by the change in metabolic gene expression resulting from *ΔpurR *mutation. The level of cellular ATP and NAD^+^/NADH ratio are factors that could influence the induction of the reactive oxygen species following accumulation of the topoisomerase cleavage complex [7,8].

FNR is a global regulator for the response of many genes to oxygen level [22,28]. It can activate or repress different genes directly by binding to the upstream regulatory region [19]. FNR also activates the transcription of the small non-coding RNA FnrS which negatively regulates the expression of multiple genes, including many that encode enzymes with functions linked to oxidative stress [26,27]. The presence of its binding site on pInter was responsible for part of the resistance to topoisomerase I cleavage complex mediated cell killing conferred by this high copy number plasmid. The oxygen level in the culture decreased as cell growth approached stationary phase even with shaking, probably resulting in partial activity of the FNR protein. Regulatory effect of FNR on transcription of acetyl coenzyme A synthetase gene in *E. coli *has been previously observed under conditions that are not strictly anaerobic [30]. We showed that the protective effect of the *Δfnr *mutation on cell death following topoisomerase I cleavage complex accumulation was more prominent under low oxygen condition, consistent with the increased activity of FNR expected when oxygen is limiting. FNR may influence cell death pathway initiated by topoisomerase cleavage complex by suppressing the genes that can enhance the response to reactive oxygen species implicated in the cell death pathway. Alternatively, decrease in FNR activity may alter the metabolic state of the cell, so that it is less susceptible to the oxidative damage cell death pathway.

In future studies, it would be informative to express FNR and/or PurR in the corresponding deletion mutants under the control of an inducible promoter. This would allow examination of promoter occupation across the genome and correlate global gene expression pattern with sensitivity to the oxidative damage cell death pathway.

## Methods

### Bacterial strains and plasmids

Genomic DNA *E. coli *strain YT103 was used to generate the chromosomal fragment library. It has *ydeA*::kan and Δ*ara *mutations to avoid having clones in the library that are known to decrease expression from the arabinose inducible BAD promoter [31]. Sensitivity to topoisomerase I cleavage complex mediated cell death was measured in *E. coli *strain BW27784 and its derivatives. This genetic background allows uniform expression of recombinant mutant topoisomerase I under the control of the BAD promoter in response to arabinose [32]. The YpTOP1-D117N clone with the highly lethal Asp to Asn mutation at the first aspartate of the TOPRIM DxDxxG motif [33] was integrated into the chromosome in strain BW117N [10]. Mutant YpTOP1 with the Gly to Ser mutation at position G122S of the TOPRIM motif was expressed from plasmid pAYTOP128 [11]. Other chromosomal mutations were introduced into *E. coli *BW27784 by P1 transduction. PCR amplification of specific *E. coli *chromosomal fragments for cloning, and site-directed mutagenesis of the plasmid encoded sequence, were carried out using *Pfu II *Ultra DNA polymerase (from Stratagene). All strains and plasmids used in this study are listed in Table 4. LB medium was used for culture unless otherwise stated.

**Table 4:** *E. coli *strains and plasmids used in this study

	Relevant genotype	Source or construction
*E. coli *strains		

BW27784	Δ(*ara*BAD)*567 *Δ(*rha*BAD)*568*	Yale *E. coli *Genetic Stock Center

	Δ(*araFGH*) Φ(Δ*araEp*P_CP18_-*araE*)	[32]

BW117N	BW27784 with chromosomally integrated YpTOP1-D117N gene	[10]

AQ4335	Δ*ara leu*7697 NBRP	NBRP-*E. coli *at NIG

FB20344	MG1655 *ydeA*::Tn5KAN-I-SceI	U. Wisconsin [34]

YT103	AQ4335 *ydeA*::Tn5KAN-I-SceI	P1(FB20344) × AQ4335, Kan^r^

JW1328-1	Δ*fnr771*::kan	Yale *E. coli *Genetic Stock Center [35]

JW1650-1	Δ*purR746*::kan	Yale *E. coli *Genetic Stock Center [35]

IFL6	BW27784 Δ *fnr771*::kan	P1(JW1328-1) × BW27784, Kan^r^

IFL7	BW27784 Δ *purR746*::kan	P1(JW1650-1) × BW27784, Kan^r^

Plasmids		

pAYTOP128	Mutant derivative of pAYTOP encoding YpTOP1 with G122S, M326V and A383P mutations	[11]

pCRII	High copy number cloning vector	Invitrogen

pAQ5	pCR-XL-TOPO cloning product of *E. coli *chromosome fragment 2618398-2620765	This study

pAQ5-1	pCR-XL-TOPO carrying *upp *gene and the intergenic region of *upp-purMN*	This study

pAQ5-2	pCR-XL-TOPO carrying *purM *gene and the intergenic region of *upp-purMN*	This study

pInter	pCR-XL-TOPO carrying the intergenic region of *upp-purMN*	This study

pInterD1	pInter with the FNR binding site deleted	This study

pInterD2	pInter with the PurR binding site deleted	This study

### Screening of clones conferring resistance to topoisomerase I cleavage complex

*E. coli *YT103 chromosomal fragments, with sizes between 2.5 and 4.5 kbp, generated from partial Sau3A1 digestion and sonication were gel purified and used to generate a high copy number plasmid library with the pCR-XL-TOPO cloning system (Invitrogen). The pooled plasmid library with >10,000 genomic DNA clones was used to transform *E. coli *BW117N by electroporation. Transformants that were resistant to the dominant lethal effect of YpTOP1-D117N were selected by plating on LB plates with antibiotics and 0.002% arabinose. Plasmid was isolated from viable colonies and confirmed in subsequent transformation of BW117N to confer resistance to cell killing mediated by topoisomerase I cleavage complex accumulation.

### Cell viability assays

Transformants of BW27784 or BW117N were grown in LB medium with antibiotics to exponential phase (OD_600 _= 0.4). The cultures were treated with either arabinose to induce recombinant mutant topoisomerase I or the gyrase inhibitor norfloxacin for the stated length of time at 37°C with shaking at 215 rpm unless otherwise stated. Serial dilutions of the cultures were then plated on LB plates with antibiotics with 2% glucose added for BW117N or BW27784 transformed with pAYTOP128, and incubated overnight. The viable colony counts from the treated cultures were normalized against the untreated culture to calculate the survival ratio. The results shown represent the average and standard errors of at least three experiments.

### Western blot analysis of recombinant *Y. pestis *topoisomerase I expression

Exponential phase cultures were treated with indicated concentration of arabinose for 2 or 2.5 h. Cells were collected by centrifugation from volumes based on OD_600 _and resuspended in SDS gel sample buffer before boiling for 5 min and SDS page for total protein analysis. The coomassie blue stained gel was examined to confirm equal loading. For improved control of equal loading in experiments using minimal media, total soluble proteins were prepared and quantitated by the BioRad Dc protein assay. Mouse monoclonal antibodies against *E. coli *topoisomerase I were used in Western blot analysis to detect the highly homologous *Y. pestis *topoisomerase I. Partially degraded *Y. pestis *topoisomerase I (YpTOP*) was also detected.

### Hydroxyl radicals formation assay

BW27784 transformed with vector or pInter was grown to exponential phase in LB before treatment with 250 ng/ml norfloxacin, or left untreated as control. After the indicated time, hydroxyl radicals were measured with the fluorescent reporter dye, 3'(p-hydroxyphenyl) fluorescence (HPF) in a FACScan flow cytometer (Becton Dickinson) [13].

## Conclusions

We demonstrated that titration of the *E. coli *transcription factors FNR and PurR by plasmid clones with the transcription factor binding sites can confer resistance to cell killing mediated by mutant topoisomerase I cleavage complex and norfloxacin acting on DNA gyrase. Our study showed that perturbation of the global regulator FNR and PurR function as well as increase in purine nucleotide availability, could affect the oxidative damage cell death pathway initiated by topoisomerase cleavage complex. The metabolic state of the cell is likely to be an important factor for the bactericidal outcome in this cell death pathway.

## Authors' contributions

IL identified and characterized the relevant plasmid clones and *E. coli *mutants and participated in experimental design, data analysis and manuscript drafting. SA participated in the flow cytometry experiment, data analysis and manuscript drafting. YT conceived of the study, participated in experimental design, data analysis and manuscript drafting. Additionally, all authors have read and approved the final manuscript.
